# Enteric Fever Caused by* Salmonella enterica* Serovars with Reduced Susceptibility of Fluoroquinolones at a Community Based Teaching Hospital of Nepal

**DOI:** 10.1155/2017/2869458

**Published:** 2017-11-06

**Authors:** Anjeela Bhetwal, Anjila Maharjan, Puspa Raj Khanal, Narayan Prasad Parajuli

**Affiliations:** ^1^Department of Laboratory Medicine, Manmohan Memorial Institute of Health Sciences, Kathmandu, Nepal; ^2^Division of Clinical Microbiology, Department of Clinical Laboratory Services, Manmohan Memorial Medical College and Teaching Hospital, Kathmandu, Nepal

## Abstract

Enteric fever continues to be an important public health problem especially in developing countries of the tropical region including Nepal. In this study, we aimed to investigate the incidence of enteric fever associated with* Salmonella enterica* and determine its antimicrobial susceptibilities to therapeutic antimicrobials in a community based teaching hospital of Nepal. A total of 2,304 blood samples from suspected enteric fever patients attending Manmohan Memorial Teaching Hospital were processed with standard microbiological methods for the isolation and identification of bacterial pathogens. The* Salmonella enterica* clinical strains were subjected to antimicrobial susceptibility testing by Kirby–Bauer disk diffusion method, and the results were interpreted according to the criteria suggested by the Clinical and Laboratory Standards Institute (CLSI). A total of 245 (10.6%) cases of enteric fever associated with* Salmonella enterica* were confirmed by blood culture. Out of them, 162 (66.1%) were caused by* Salmonella* Typhi and 83 (33.9%) by* Salmonella* Paratyphi. On Kirby–Bauer disk diffusion antimicrobial susceptibility testing,* Salmonella *isolates were highly susceptible to cefixime (100%), ceftriaxone (100%), ampicillin (97.9%), cotrimoxazole (94.6%), azithromycin (96.7%), tetracycline (95.5%), and chloramphenicol (97.5%), respectively. Two hundred twenty-six (92.2%) of* Salmonella *isolates were nalidixic acid resistant with reduced susceptibility to ciprofloxacin (36.7%) and ofloxacin (54.8%), respectively. Although the rate of MDR* Salmonella* strains was very low (<5%), their reduced susceptibility to fluoroquinolones has restricted their routine empirical use. Third generation cephalosporins are the safest choice for empirical use but ampicillin, cotrimoxazole, azithromycin, and chloramphenicol can be effective alternatives.

## 1. Background

Enteric fever is a life-threatening systemic illness caused by serovars of human-adapted pathogen*, Salmonella enterica *[1]. It is an acute and invasive infection of the gastrointestinal system and causes a devastating burden in many low- and middle-income countries with significant morbidity and mortality [2, 3]. The recent global incidence of enteric fever has been reported to range from 11.9 million to 26.9 million per year with approximate case fatality rate of 1% [4, 5]. In Nepal, enteric fever or typhoid fever, commonly known as* “Bisham Jwaro”* (fever with poison), is prevalent in mountains, valleys, and southern Terai region as an endemic disease, with its peak incidence occurring from May to August. It is one of the leading diagnoses of fever in most of the hospitals in Nepal. Series of enteric fever outbreaks with variable drug susceptibilities have been reported from the country, and the vast bulk of them have been linked to fecal contamination of foods and drinking water [6, 7].

Antimicrobial agents are the mainstay of therapy in enteric fever so as to prevent the complications associated with severe illness and death of the patients [8]. However, the reduced susceptibility of* Salmonella enterica* isolates to commonly used antibiotics continues to be a major problem for effective therapy of enteric fever, particularly in developing countries [9, 10]. Multidrug resistant* Salmonella enterica* strains (resistant to chloramphenicol, ampicillin, and cotrimoxazole) are increasingly reported from Asian countries [11, 12]. In Nepal, also, there have been several enteric fever epidemics with changing antibiotic resistance patterns [13, 14] since the first report of multiple drug resistant (MDR)* S.* Typhi in 1991 [15]. Due to the emergence of MDR strains, the use of chloramphenicol, ampicillin, and cotrimoxazole has become infrequent and quinolones became the first choice for the treatment of typhoid fever in endemic areas [16]. Subsequently, during the last few years, nalidixic acid resistant strains associated with reduced susceptibility to fluoroquinolones in the patients treated with quinolones have been increasingly reported elsewhere [17] including Nepal [6, 18]. Cephalosporins and macrolides are nowadays the therapeutic choices for enteric fever cases in our region [19], but the emergence of multidrug resistant and extended spectrum *β*-lactamase (ESBL) producing strains has created a therapeutic challenge [14].

In this backdrop, when the treatment options for enteric fever are decreasing, extensive workup and evaluation of alternative choices for effective therapy and management of enteric fever cases are becoming vital. Therefore, this study was intended to determine the spectrum of* Salmonella enterica* serovars isolated from the blood culture of the patients suffering from enteric fever and their antibiotic susceptibility pattern to commonly used antibiotics in a community based tertiary care teaching hospital in Kathmandu, Nepal.

## 2. Methodology

### 2.1. Study Design and Setting

This was a laboratory-based descriptive study carried out over a period of two years (April 2015 to March 2017) in the Department of Clinical Microbiology of Manmohan Memorial Teaching Hospital (MMTH), a community based tertiary care teaching hospital with 300-patient beds in Swayambhu, Kathmandu, Nepal. The hospital is located outside the main city of Kathmandu and is a major hospital for rural villages nearby capital.

### 2.2. Inclusion and Exclusion Criteria

In this study, patients clinically suspected of enteric fever presented to our hospital were enrolled. However, the patients already on antibiotics and repeated samples from the same patient were excluded.

### 2.3. Specimen Collection, Processing, and Identification of* Salmonella* Isolates

Patients visiting outpatient departments or admitted to the inpatient units suspected of enteric fever were investigated clinically by respective unit physicians. A blood culture specimen was taken with the aseptic technique by cleansing of the collection site with 70% alcohol and subsequently followed by povidone iodine. Five milliliters (for pediatric patients) and 10 ml (for adult patients) of blood specimen were collected and inoculated into brain heart infusion (BHI) broth at the blood to broth ratio of 1 : 10. After incubation, at 37°C for 24, 48, and 72 hours, subculture was made on blood agar and MacConkey agar plates regardless of the turbidity. The plates were observed for bacterial growth after 24 hrs of aerobic incubation at 37°C.* Salmonella enterica *isolates were identified using standard microbiological techniques: biotyping (colony morphology, staining reaction, and biochemical characteristics) and serotyping using specific antisera (Denka Seiken Co. Ltd., Tokyo, Japan) [20]. Samples were considered sterile if no growth was observed on subculture after 7 days of aerobic incubation at 37°C. Patient information, that is, patient name, age, sex, ward/bed number (if admitted), brief clinical history, duration of hospital stay, and history of antibiotic use, was taken.

### 2.4. Antimicrobial Susceptibility Testing

Antimicrobial susceptibility testing of the isolated strains of* Salmonella enterica* was carried out using the disk diffusion method (modified Kirby–Bauer method) on Mueller–Hinton agar (HiMedia, India) following standard procedures recommended by the Clinical and Laboratory Standards Institute (CLSI), Wayne, USA [21]. We analyzed the susceptibility of common therapeutic antimicrobial agents including ampicillin (10 *μ*g), nalidixic acid (30 *μ*g), ofloxacin (5 *μ*g), ciprofloxacin (5 *μ*g), chloramphenicol (30 *μ*g), cotrimoxazole (25 *μ*g), cefixime (30 *μ*g), ceftriaxone (30 *μ*g), cefotaxime (30 *μ*g), azithromycin (15 *μ*g), and tetracycline (30 *μ*g) (HiMedia Laboratories, India). The results of the antibiotic susceptibility were determined on the basis of interpretative zone diameters suggested by CLSI [21]. For standardization,* Escherichia coli* ATCC-25922 was used as the control organism for antibiotic sensitivity.

### 2.5. Statistical Analysis of Data

Data regarding the bacterial isolates, their susceptibility to various antibiotics, and other information were entered and analyzed using the Statistical Package for Social Sciences (SPSS™) version 20.0 (IBM, Armonk, NY, USA). The results are presented in percentage-based distribution.

### 2.6. Ethical Approval

Written approval (Ref 009/MMIHS/2072) was obtained from Institutional Review Committee of Manmohan Memorial Institute of Health Sciences (MMIHS) before starting the study. Written informed consent was taken from every patient or their guardians before enrollment into the study.

## 3. Results

### 3.1. Patients with Enteric Fever and Their Demographics

During the study period, out of a total of 2,304 blood culture specimens from the patients suspected with enteric fever, 245 (10.63%) were found positive for the growth of* Salmonella enterica*, confirming the enteric fever. More samples were from outpatients (1701, 73.8%) as compared to inpatients (603, 26.2%). Male patients (149, 60.8%) constitute the major subgroup affected with enteric fever, and* Salmonella enterica* serovar Typhi (162, 66.1%) was the common serovar associated with enteric fever in our setting. In addition, the proportion (178, 72.6%) of enteric fever cases occurred in patients of the age group of 15–44 years was higher than any other age group (Table 1).

### 3.2. Antimicrobial Susceptibilities of* Salmonella* Serovars Typhi and Paratyphi

Table 2 illustrates the susceptibilities of* Salmonella enterica* serovars Typhi and Paratyphi in our study. Overall, 39.0% and 46.9% of* Salmonella* Typhi and Paratyphi serovars were susceptible to ciprofloxacin, while 55.0% and 65.0% of them were susceptible to ofloxacin. Reduced susceptibility to ciprofloxacin (2.4% and 10.9% resistant, 58.6% and 42.2% intermediate susceptible to Typhi and Paratyphi, resp.) and ofloxacin (5.0% and 8.4% resistant, 40.0% and 26.6% intermediate susceptible to Typhi and Paratyphi, resp.) was observed. Other than fluoroquinolones, the overall susceptibility of* Salmonella* isolates to chloramphenicol, ampicillin, cotrimoxazole, and azithromycin was found to be excellent, that is, 97.5%, 97.9%, 94.6%, and 96.7% each, suggesting revival of conventional antibiotics in our setting.

### 3.3. Nalidixic Acid Resistant* S.* Typhi and* S.* Paratyphi

Nalidixic acid resistance was very much common among the isolates of* Salmonella enterica*. Overall, 92.2% of the isolates were NA resistant. Although statistically nonsignificant (*p* > 0.05),* S.* Paratyphi strains showed higher rate (97.6%) of NAR than* S*. Typhi (89.5%) (Table 3).

## 4. Discussion

Enteric fever remains a major cause of febrile illness in the urban areas of endemic countries with limited water and sanitation infrastructure [22]. World Health Organization (WHO) has recommended vaccination with existing Vi polysaccharide vaccine targeting high-risk areas of typhoid fever [16]. Besides, estimation of the disease burden and its etiology along with antimicrobial susceptibilities would be helpful in the development of effective prevention and control interventions. Nepal is a pocket area of typhoid endemicity due to the poor sanitation status and cross-contamination of food and drinking water with sewage [7].

Overall, the incidence rate of enteric fever caused by serovars of* Salmonella enterica *in our hospital was 10.6%. The finding of our study is similar to the reports of Sharma et al. (8.9%) [23], Shrestha et al. (13.3%) [24], and Easow et al. (15.6%) [25] from nearby hospitals of Nepal. The lower rates of blood-culture-positive enteric fever might also be due to the use of antibiotics prior to the blood culture and low blood volume used for culture (10 ml for adult and 5 ml for children) as well as self-medication before arrival to the hospital. However, we did not evaluate the prior antibiotic consumption by the patients before enrollment. In addition, our data is well supported by recent epidemiological studies in Nepal [26, 27], where the great bulk of undifferentiated febrile illness was associated with other atypical organisms [27]. In addition, male patients (149, 12.5%) and patients in the 15–44 age group (178, 72.6%) showed higher incidence of enteric fever cases, corroborating the previous reports from Nepal [19, 28]. Probable reason behind this variation might be regular eating-out habit of males and the adult age population consuming infected food and water from restaurants.

Out of 245 culture-confirmed enteric fever cases, 162 (66.1%) were caused by* Salmonella* Typhi and 83 (33.9%) were by* Salmonella* Paratyphi. The dominance of Typhi serovars in enteric fever in our study complies with the observation made by Adhikari et al. (64.1% and 35.9% of the* S*. Typhi and* S. *Paratyphi, resp.) [28]. However, Shirakawa et al. documented* S. *Paratyphi as more prevalent serovar in Kathmandu, Nepal [18], which is supported by another recent study of Pramod et al. (35.9%* S.* Typhi and 64.1%* S.* Paratyphi) [29]. Although, there is no such well-established cause for serovar variation in enteric fever cases, higher incidence of Typhi might be due to waterborne transmission of* S.* Typhi as it usually involves smaller inocula than paratyphoid achieved through food borne transmission that requires large inocula [30].

Fluoroquinolones (FQs), ciprofloxacin and ofloxacin, are the mainstay of therapy against* Salmonella* infections because they are available for oral use and are also less expensive options [31]. However, they are increasingly becoming ineffective in enteric fever cases due to the emergence of nalidixic acid resistant (NAR) strains [12, 13]. In our study, rate of NAR, a phenotypic marker for reduced susceptibility to fluoroquinolones, was very high (92.2%). Moreover,* S.* Paratyphi strains showed even higher rate (97.6%) of NAR than* S*. Typhi (89.5%). Similar rates of resistance to nalidixic acid (NA) among* S*. Paratyphi isolates were reported in the studies of Acharya et al. [30], Shirakawa et al. [18], and Chand et al. [19] from Nepal. The rate of resistance to NA we found is high when compared to the reports of Shrestha et al. (83.1%) [24] from Nepal and Kadhiravan et al. from India (78%) [32]. The high rate of resistance to NA and emergence of strains with full resistance to FQs by* Salmonella* spp constitute a major problem in Nepal [13]. On the other hand, the main cause of resistance to quinolones in Gram-negative bacteria, including* Salmonella*, is the mutation in the genes coding for DNA gyrase* (gyrA and gyrB)* and topoisomerase IV* (parC and parE)* [33]. Enhanced active efflux and early overproduction of the* AcrA* pump in isolates with the* gyrA* mutation could be responsible for the decrease in susceptibility to FQs. Low level of resistance to ciprofloxacin, probably due to the point mutation in the* gyrA* gene, may not be detected by in vitro susceptibility tests using the current MIC breakpoints for ciprofloxacin. Therefore, in vitro resistance to NA can be used to detect this low level resistance [31, 34]. However, susceptibility testing generally adopted in the resource-poor laboratories of developing countries including Nepal is limited to disk diffusion technique which may not be adequate to determine reduced susceptibility to FQs [12, 24].

Furthermore, the increasing number of isolates having reduced susceptibility to ciprofloxacin and ofloxacin may be due to irrational use of FQs, as the empirical antimicrobial agent, and emergence of NAR strains [31]. Besides, for NAR strains, reduced susceptibility to ciprofloxacin (31.7% and 45.6% susceptible, 65.6% and 43.3% intermediate susceptible, and 2.7% and 11.1% resistant in Typhi and Paratyphi, resp.) and ofloxacin (49.7% and 64.2% susceptible, 44.8% and 27.1% intermediate susceptible, and 5.6% and 8.7% resistant in Typhi and Paratyphi, resp.) was found. In NAS* Salmonella *Typhi and Paratyphi isolates, sensitivity to fluoroquinolones reached 100%. Reduced susceptibilities to fluoroquinolones were recorded previously for this region [28, 29, 35]. However, 100% susceptibility of* Salmonella* Typhi and 96.7% susceptibility of* Salmonella* Paratyphi to ciprofloxacin were observed in a study by Chand et al. [19]. In our study, the isolates with reduced fluoroquinolones susceptibility were also uniformly resistant to NA. NA susceptibility showed a predictive value of 100% for ciprofloxacin and ofloxacin susceptibility, whereas NA resistance showed a predictive value of 58.95% for ciprofloxacin and ofloxacin resistance.

Besides fluoroquinolones, the overall susceptibility of* Salmonella* isolates to chloramphenicol was found to be 97.5% (96.3% for* S*. Typhi and 100% for* S. *Paratyphi). Susceptibility of* Salmonella* isolates to other first-line drugs, that is, ampicillin, cotrimoxazole, and azithromycin, was also excellent, 97.9%, 94.6%, and 96.7%, respectively. Chloramphenicol was once considered the drug of choice for enteric fever acquired resistance within few years of its introduction, but later, MDR* Salmonella* resistant to chloramphenicol, ampicillin, and trimethoprim sulfamethoxazole emerged in the late 1980s and early 1990s [36, 37]. Reemergence of susceptibility to chloramphenicol and other first-line drugs in previously resistant areas has been reported in studies done earlier [19, 24, 38–40]. The decreased use of first-line antibiotics in treating* Salmonella* and other infections could be likely the reason for this reemergence of susceptibility [24]. Cephalosporins (ceftriaxone, cefotaxime, and cefixime) exhibited excellent efficacy towards isolated* Salmonella *serovars with 100% sensitivity. Therefore, oral cephalosporins and macrolides are the first-line agents for empirical treatment of enteric fever cases [4, 41]. Fluoroquinolones would still be the effective therapeutic regimen in our scenario because a good proportion of quinolones is found susceptible, but susceptibility test should be performed before starting the quinolone therapy.

### 4.1. Limitation of the Study

Although this was a hospital-based study, we could not evaluate the risk factors and outcomes of enteric fever in our setting. Underequipped laboratories, lack of resources for molecular techniques, limited options for antimicrobial susceptibilities are the main drawbacks to high-quality data for clinical evaluation. This is a single-center study; multicenter cohort study including national wide geographical area would have generated more significant results in our country.

## 5. Conclusion

Sensitivity pattern of the* Salmonella* isolates is changing especially in the community settings with reemergence of ampicillin, cotrimoxazole, and chloramphenicol sensitive* Salmonella* strains. Fluoroquinolones are becoming less effective because of haphazard use and are no more suitable for empirical use in our settings. Use of different antibiotic agents as per the sensitivity patterns and disease severity can help to decrease excessive use of cephalosporin and macrolide and ultimately help to effectively delay the emergence of resistance to these agents. Only good foresight and proper antibiotic policy can save our communities from the inevitable burden of antibiotic resistance.

## Figures and Tables

**Table 1:** Patients with enteric fever and their demographics.

Age group (years)	Enteric fever cases			
	Total (%)	Confirmed (%)	*Salmonella* Typhi (%)	*Salmonella* Paratyphi (%)
<5	261 (11.3)	17 (6.5)	11 (64.7)	6 (35.3)
5–14	355 (15.4)	37 (10.4)	22 (59.4)	15 (40.6)
15–44	1,210 (52.2)	178 (14.7)	119 (66.8)	59 (33.2)
45–59	281 (12.2)	8 (2.9)	6 (75.0)	2 (25.0)
≥60	197 (8.5)	5 (2.5)	4 (80.0)	1 (20.0)

*Total*	*2,304*	*245 (10.6)*	*162 (66.1)*	*83 (33.9)*

**Table 2:** Antimicrobial susceptibilities of *Salmonella enterica* serovars.

Antibiotics	*Salmonella enterica* serovar Typhi (*n* = 162)			*Salmonella enterica* serovar Paratyphi (*n* = 83)		
	*S* (%)	*I* (%)	*R* (%)	*S* (%)	*I* (%)	*R* (%)
Ampicillin	158 (97.6)	0 (0)	4 (2.4)	82 (98.8)	0 (0)	1 (1.2)
Nalidixic acid	17 (10.5)	0 (0)	145 (89.5)	2 (2.4)	0 (0)	81 (97.6)
Ciprofloxacin	63 (39.0)	95 (58.6)	4 (2.4)	39 (46.9)	35 (42.2)	9 (10.9)
Ofloxacin	89 (55.0)	65 (40.0)	8 (5.0)	54 (65.0)	22 (26.6)	7 (8.4)
Cotrimoxazole	151 (93.2)	0 (0)	11 (6.8)	81 (97.6)	0 (0)	2 (2.4)
Cefixime	162 (100)	0 (0)	0 (0)	83 (100)	0 (0)	0 (0)
Cefotaxime	162 (100)	0 (0)	0 (0)	83 (100)	0 (0)	0 (0)
Ceftriaxone	162 (100)	0 (0)	0 (0)	83 (100)	0 (0)	0 (0)
Azithromycin	156 (96.3)	0 (0)	6 (3.7)	81 (97.6)	0 (0)	2 (2.4)
Tetracycline	154 (95.1)	0 (0)	8 (4.9)	80 (96.3)	0 (0)	3 (3.7)
Chloramphenicol	156 (96.3)	0 (0)	6 (3.7)	83 (100)	0 (0)	0 (0)

*S*: sensitive, *I*: intermediate sensitive, *R*: resistant.

**Table 3:** Distribution of nalidixic acid (NA) resistant *S. *Typhi and *S. *Paratyphi.

Serovars	Nalidixic acid		*p*
	Resistant (%)	Sensitive (%)	
*S*. Typhi	145 (89.5)	17 (10.5)	0.624
*S.* Paratyphi	81 (97.6)	2 (2.4)	
Total	226 (92.2)	19 (7.8)	

## Abbreviations

ASM: American Society for Microbiology
ATCC: American type culture collection
BHI: Brain heart infusion broth
CLSI: Clinical and Laboratory Standard Institute
FQs: Fluoroquinolones
MDR: Multidrug resistant
NARS: Nalidixic acid resistant* Salmonella*
S. Typhi: *Salmonella enterica* subspecies enterica serovar Typhi
S. Paratyphi: *Salmonella enterica* subspecies enterica serovar Paratyphi
ZOI: Zone of inhibition.
